# Integrating ESM‑2 and Graph Neural Networks with AlphaFold‑2 Structures for Enhanced Protein Function Prediction

**DOI:** 10.1021/acsomega.5c05484

**Published:** 2025-08-16

**Authors:** Thi-Tuyen Nguyen, Zhuocheng Jiang, Van-Nui Nguyen, Nguyen Quoc Khanh Le, Matthew Chin Heng Chua

**Affiliations:** † University of Information and Communication Technology, Thai Nguyen University, Thai Nguyen 25000, Viet Nam; ‡ Department of Biomedical Informatics, Yong Loo Lin School of Medicine, 37580 National University of Singapore, Singapore 119228, Singapore; § In-Service Master Program in Artificial Intelligence in Medicine, College of Medicine, 38032 Taipei Medical University, Taipei 110, Taiwan; ∥ AIBioMed Research Group, Taipei Medical University, Taipei 110, Taiwan; ⊥ Translational Imaging Research Center, Taipei Medical University Hospital, Taipei 110, Taiwan

## Abstract

Protein function prediction is essential for elucidating biological processes and accelerating drug discovery. However, the vast number of unannotated protein sequences and the limited availability of experimentally validated functional data remain major challenges. Although deep learning models based on protein sequences or protein–protein interaction networks have shown promise, their performance is still restricted, particularly for proteins without interaction data. Furthermore, many existing approaches treat sequence and structural information separately, potentially resulting in suboptimal feature representations. To address these limitations, we propose an improved graph-based framework that integrates two key innovations: (i) ESM-2, a state-of-the-art protein language model, to generate semantically rich sequence embeddings; and (ii) a hybrid pooling mechanism within graph convolutional blocks to better capture both global and local structural features from AlphaFold2-predicted structures. Experiments on the human proteome demonstrate that our model consistently outperforms existing methods in predicting molecular function, cellular component, and biological process annotations. These findings highlight the advantages of combining advanced sequence representations with enhanced structural learning for accurate and generalizable protein function prediction.

## Graphical abstract

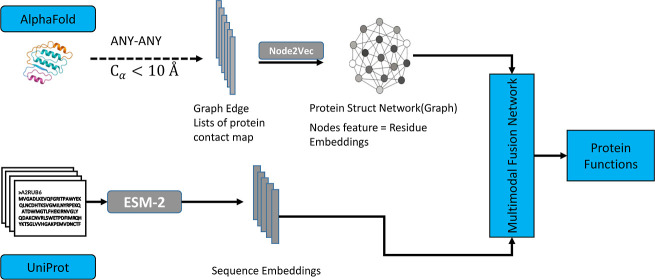

## 1 Introduction

Protein function prediction is a fundamental task in computational biology, as proteins play central roles in virtually all cellular processes, including catalysis, molecular transport, signal transduction, and gene regulation.^1,2^ Accurate functional annotation of proteins is therefore critical for understanding biological mechanisms and supporting applications such as drug discovery, disease diagnosis, and synthetic biology.^3^ However, experimental techniques such as enzyme assays, surface plasmon resonance, and gel electrophoresis, while accurate, are labor-intensive, costly, and impractical for large-scale annotation.

The rapid growth of protein databases—exemplified by UniProt, which contains over 100 million sequences but only a small fraction with experimentally verified functions^4^—highlights the urgent need for automated, scalable computational methods. The gene ontology (GO) framework,^5^ which categorizes protein functions into biological processes, molecular functions, and cellular components, has become the standard for functional annotation.^6^ Traditional sequence alignment tools such as BLAST^7^ and HMMER,^8^ provide valuable insights but often fail to detect functional divergence among structurally distinct yet sequence-similar proteins.

Machine learning, particularly deep learning, has emerged as a powerful alternative. Sequence-based deep learning models leverage architectures such as convolutional neural networks (CNNs), recurrent neural networks (RNNs), and transformers to extract functional signals directly from protein sequences.^9,10^ For instance, DeepGO^11^ uses CNNs and bidirectional long short-term memory (BiLSTM), while models such as ProteinBERT^12^ and evolutionary scale modeling (ESM)^13^ employ large transformer-based encoders trained on massive protein sequence data sets. Despite these advances, sequence-only models frequently struggle to distinguish proteins with similar sequences but divergent functions, underscoring the importance of incorporating structural information.

Protein three-dimensional (3D) structures provide critical insights into biochemical activity and molecular interactions. Traditional structure-based methods such as TM-align^14^ and DALI^15^ align query proteins to annotated templates to infer function, improving accuracy but suffering from high computational costs and reliance on experimentally determined structures. The advent of AlphaFold2 ^16^ has largely overcome this barrier by enabling accurate, large-scale structural predictions, creating new opportunities for structure-aware functional annotation.

Graph-based deep learning methods, particularly graph convolutional networks (GCNs),^17^ offer a natural framework for leveraging structural information. By modeling proteins as contact graphs, where residues are nodes and edges represent spatial proximity, GCNs can effectively capture geometric and topological information. These models have been successfully applied to tasks such as protein surface analysis,^18^ protein–protein interaction prediction,^19^ and model quality assessment.^20^

For protein function prediction specifically, models such as DeepFRI^21^ and HEAL^22^ have demonstrated the value of combining structural features with deep learning. HEAL, for example, integrates AlphaFold2-predicted structures^16^ and language model embeddings using hierarchical graph transformers and contrastive learning, showcasing the potential of multimodal approaches. However, such models remain isolated examples; most existing methods still treat sequence and structure separately or rely on shallow feature concatenation, which fails to fully exploit the complementary nature of these modalities. This lack of deep integration limits their ability to learn enriched and discriminative representations.

Moreover, few studies provide clear architectural designs for systematically unifying sequence and structure information. Addressing this gap is the central motivation for our work: to develop an end-to-end multimodal framework that jointly learns from both sequence-level and structure-level inputs to improve predictive accuracy and generalization across diverse protein families.

Building upon the Struct2GO framework,^23^ we introduce several key innovations. First, we replace SeqVec with ESM-2 to derive context-rich sequence embeddings. Second, we use Node2Vec^24^ to encode residue-level structural relationships as informative graph representations. Third, we employ a hybrid pooling strategy combining SAGPool and MixedPooling to capture multiscale structural features. These components are integrated into a unified, computationally efficient architecture.

Our proposed framework (Figure 1) embeds protein sequences using ESM-2 and converts predicted structures into Node2Vec-encoded contact graphs.^24^ These representations are processed via dual branches—a hybrid-pooled GCN for structure and a CNN for sequence—followed by a transformer encoder for multilabel GO term classification.

**Figure 1:**
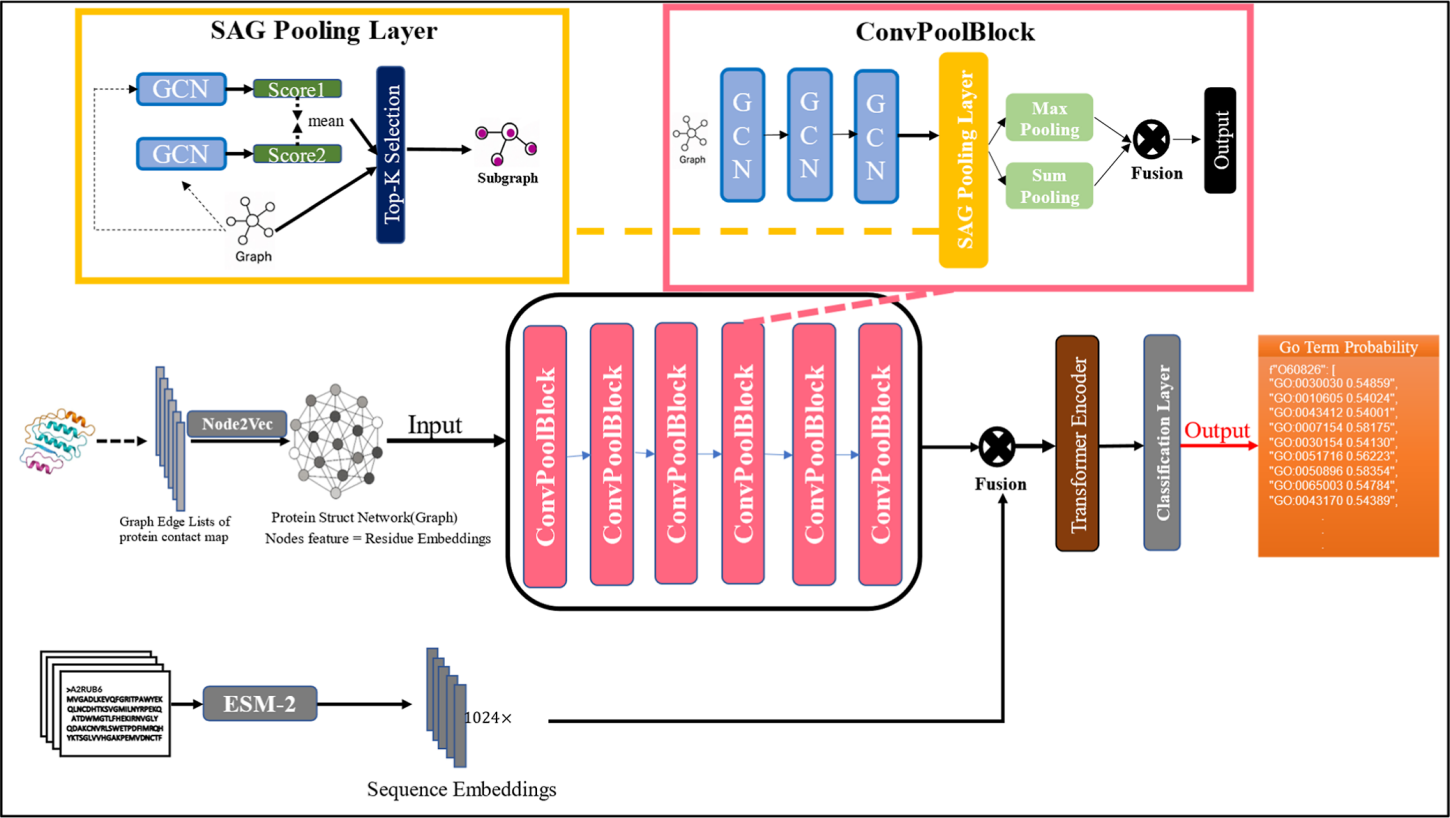
Model structure.

This study addresses the limitations of previous approaches^23^ by improving both sequence representation and structural feature learning. Our experiments demonstrate that the proposed multimodal framework significantly outperforms benchmark methods, providing a robust and scalable solution for large-scale protein function prediction.

Finally, while multimodal integration introduces additional computational cost, we mitigate this by adopting practical architectural choices, such as using a moderate-sized ESM-2 variant, precomputing embeddings, and maintaining manageable model depth. These design strategies strike a balance between performance and efficiency, enabling our approach to be applied at proteome scale.

## 2 Materials and Methods

### 2.1 Data Collection

This study utilizes two primary types of data: protein structural information and protein sequence data. For the development and evaluation of our enhanced model, protein structural data in PDB format were extracted from the AlphaFold2-predicted structures of the human proteome. Corresponding protein sequences were obtained from UniProt,^4^ while functional annotations were sourced from the gene ontology annotation (GOA) project and ontology definitions from the official GO database.

In total, the data set comprises 20,504 proteins annotated with GO terms from the three major ontological categories: molecular function ontology (MFO), biological process ontology (BPO), and cellular component ontology (CCO). GO term mappings were parsed using the go.obo file to maintain consistency with the hierarchical structure of the ontology. To ensure label quality and reduce noise, rare GO terms with low frequency were excluded based on a predefined threshold. After filtering, the resulting data set contained 308 terms for MFO, 310 for CCO, and 713 for BPO.

For benchmarking, we reproduced the original model using the same training, validation, and test splits provided by the original authors. These data sets were preprocessed and partitioned according to their published methodology. The baseline model was retrained using the publicly available source code and hyperparameter configurations reported in the Struct2GO publication.^23^

### 2.2 Protein Representation

Protein representation is achieved by integrating both structural and sequence-based information. Structural data are modeled using contact maps, where amino acids are represented as nodes, and their embeddings are computed using the Node2Vec algorithm.^24^ For sequence data, embedding vectors are derived from pretrained protein language models.

#### 2.2.1 Graph Construction

Amino acids within a protein are connected by peptide bonds; to model these relationships, protein structure graphs are commonly constructed to facilitate computational analysis and serve as input for deep learning models. In this work, we construct individual graphs based on structural data predicted by AlphaFold-2,^16^ with each node representing a single amino acid residue.

To capture spatial relationships between residues, we employ the Any–Any method for graph construction. This approach considers distances between all atomic pairs of two amino acids. Following a widely accepted convention, an edge is established between two nodes if the distance between their respective Cα atoms is less than 10 Å. This strategy ensures the preservation of key spatial interactions in the three-dimensional structure, including those between residues that are sequentially distant but spatially proximal due to protein folding.

Representing protein structures as graphs is a critical preprocessing step that facilitates the extraction of spatial features, enhancing the performance of deep learning models in protein function prediction tasks. The resulting contact maps, derived from the constructed edge lists, transform the complex 3D protein structure into a tractable 2D representation while retaining essential geometric information. Among various methods proposed for contact map construction, the Any–Any method was selected for its superior ability to capture fine-grained spatial relationships within protein structures.

#### 2.2.2 Residue-Level Representation

Accurately representing the spatial features of amino acid residues within a protein requires more than simple one-hot encoding, as it cannot capture the complex three–dimensional relationships between residues.^25^ To address this limitation, graph-based representation techniques are widely employed, as they enable the extraction of spatially aware embeddings for each node (i.e., residue) in the protein graph.

In this study, we utilize Node2Vec^24^ to generate residue-level embeddings from the constructed protein graphs. Node2Vec is a network embedding algorithm based on a biased random walk strategy, designed to efficiently explore the graph structure and learn continuous feature representations of nodes. The algorithm performs multiple random walks to sample node sequences, akin to word sequences in natural language processing, and then maximizes the conditional probability between a target node and its surrounding nodes to learn the embedding space.

Node2Vec introduces two hyperparameters, *p* and *q*, which govern the behavior of the random walks. Parameter *p* controls the likelihood of returning to the previous node, influencing the preference for depth-first search (DFS). Meanwhile, *q* governs the probability of visiting a new node, modulating the exploration between local (BFS-like) and global graph structures. In our implementation, we adopt *p* = 0.8 and *q* = 1.2, consistent with the original Struct2GO setting, to ensure fair comparison and reliable graph embedding quality.

The learning objective of Node2Vec is presented as follows

1 $$\max_{f}\sum_{u\in V}\log P_{r}\left(N_{S}\left(u\right)|f\left(u\right)\right)$$

In this context, *N*_S_(*u*)­denotes the set of neighboring nodes of node *u*, determined through a random walk process, and *f*(*u*) represents the feature of node *u*. The original Node2Vec paper employs a negative sampling approximation to optimize the objective function, thereby improving computational efficiency. The probability of transitioning to a specific node during the random walk is defined in the original paper as follows

2 $$P\left(c_{i}=x|c_{i-1}=v\right)=\{\begin{array}{l} \frac{\pi_{vx}}{Z}if\left(v,x\right)\in E\\ 0\;other\;wise \end{array}$$

In this formula, *Z* is a normalized constant and π_*vx*_ is the un-normalized probability of wandering from *v* to *x*, which is calculated as follows

3 $$\pi_{vx}=\alpha_{pq}\left(t,x\right)\cdot W_{vx}$$

*W*_vx_ is the weight of the edge between *v* and *x*, α_*pq*_(*t*,*x*) is adjusted based on the graph shortest path distance *d*_*tx*_ from *t* to *x*, which is calculated as

4 $$\alpha_{pq}\left(t,x\right)=\{\begin{array}{l} \frac{1}{p}\;\text{if}\;d_{tx}=0\\ 1\;\text{if}\;d_{tx}=1\\ \frac{1}{q}\;\text{if}\;d_{tx}=2 \end{array}$$

By applying the Node2Vec algorithm to the residue-level protein graph, we obtain informative and spatially contextual embeddings for each amino acid. These embeddings provide a compact and discriminative feature representation of the protein’s three-dimensional structure, thereby enhancing the capacity of downstream deep learning models to accurately predict protein function.

#### 2.2.3 Sequence Embeddings

To characterize protein sequence data, we employ both one-hot encoding and embeddings derived from a pretrained protein language model. Recent advances in natural language processing (NLP)-based protein language models have shown strong performance across various protein analysis tasks. Among these, ESM-2,^13^ developed by Meta AI, is one of the most advanced models, built upon the transformer architecture. ESM-2 generates high-resolution embeddings that have been successfully applied to protein structure prediction and functional annotation.

In this study, we focus on generating protein sequence embeddings using the ESM-2 model. ESM-2 is trained on large-scale protein data sets, including UniRef50 and UniRef90, and employs a deep Transformer encoder composed of multiple self-attention layers and feedforward neural networks (FNNs). The core self-attention mechanism is defined as

5 $$Attn\left(Q,K,V\right)=soft\max\left(\frac{QK^{T}}{\sqrt{d_{k}}}\right)V$$

where the input embeddings are differentially linearly transformed to obtain Q,K,V, and the dimensions of the keys are *d*_*k*_. The masked language model (MLM) is then utilized to train by randomly overlaying some of the sequences, with a loss function using a cross entropy, defined as

6 $$L=-\frac{1}{M}\sum_{i\in masked}\log P\left(S_{i}|S_{context}\right)$$

where the number of masked positions is *M*, and *S*_context_ refers to the sequence before and after masking. Assuming the protein sequence is *S* with a length of *L*, each amino acid *S*_*i*_ is mapped to its initial embedding *e*_*i*_. Subsequently, the final sequence embedding *h*_*i*_ is obtained through multilayer Transformer processing, incorporating richer contextual information. Finally, for the entire sequence, the global embedding *h*_global_of the corresponding protein is derived by applying average pooling over the embeddings at each position.

In our experiments, we use the *esm2_t33_650M_UR50D* model provided by the official ESM API to extract sequence embeddings. This variant of ESM-2 contains 33 Transformer layers and produces embeddings with a dimensionality of 1024. All computations were performed on an NVIDIA A10 GPU using FP32 precision. We selected the *esm2_t33_650M_UR50D* model as it provides an effective balance between computational efficiency and predictive performance. While larger models such as ESM-2 3B or 15B may yield marginal improvements in accuracy, they require significantly more memory and training time, which becomes impractical for large-scale experiments. The 650 M variant was thus chosen to ensure feasibility while still capturing rich contextual information from protein sequences.

### 2.3 Model and Network

Protein function prediction is essentially a multilabel classification problem, in which the model is expected to output multiple GO terms corresponding to a single protein. The model employed in this paper adopts a hierarchical self-attention graph pooling network architecture. Specifically, the model takes as input the protein structure contact map, where the node features are the embeddings of amino acid residues. The convolutional layers then perform feature extraction and pooling. Next, the features extracted from the graph are fused with the global sequence features generated by the ESM-2 model. The fused features are further processed through a transformer encoder. Finally, the output is passed through a fully connected classification layer to predict the protein functional labels.

#### 2.3.1 Convolutional Layer

In this stage, the main task is the learning and pooling of protein structural features. Specifically, the protein contact map with added self-loops and the embeddings of its nodes are input, along with labels input into the constructed label network. The goal is to aggregate the information on similar nodes at the protein structural level and update the features for downstream classification. The graph convolutional layer used in this project is the graph convolutional layer (GraphConv),^17^ whose theoretical basis is the approximation of spectral graph convolution, allowing the extension of convolution operations to graph data structures. The forward propagation of the GraphConv layer is as follows

7 $$H^{\left(l+1\right)}=\sigma \left({\mathrm{D̃}}^{-1/2}Ã{\mathrm{D̃}}^{-1/2}H^{\left(l\right)}W^{\left(l\right)}\right)$$

where *H* ^(*l*)^ is the node feature matrix of layer *l* and has size *N* × *D*_*l*_ (number of nodes × layer *l* feature dimension), *W* ^(*l*)^is the weight of layer *l*, *A* is the adjacency matrix, *Ã* is the adjacency matrix after adding the self-loop, *D̃* is the degree matrix of *Ã*. Within the activation function is the use of the degree matrix to first normalize the self-loop adjacency matrix, weighted average of the features of each neighbor node, and then multiplied with the feature matrix of the current layer as well as the weight matrix through the activation function to obtain the next layer. This paper utilizes the interface within Deep Graph Library^26^ to implement the GraphConv layer, which is implemented in the form of a 3-layer GraphConv in the main convolution module.

#### 2.3.2 Self-Attention Graph Pooling Layer

The self-attention mechanism is widely used in deep learning and is highly effective for many problems, especially those involving ordered and complex features, as it enhances the model’s ability to capture local features and contextual understanding. In this paper, we adopt self-attention graph pooling (SAGPool)^27^ combined with a top-k node selection algorithm to construct the self-attention graph pooling layer. SAGPool is a pooling method based on the self-attention mechanism, which reduces the training burden and the size of the graph while retaining key topological and edge features as much as possible in the classification task of complex graph structures. The first step of SAGPool is to compute the attention score z on the input of the graph, which is performed as follows

8 $$z=\sigma \left(D^{-1/2}ÃD^{-1/2}X\theta \right)$$

*Ã* is the neighborhood matrix with added self-loop, *D* is the degree of this neighborhood matrix, *X* is the node features, and θ is a one-dimensional weight vector. The nodes of top-k are selected based on the mean value obtained from the self-attention score after two passes through GCN. In the node selection algorithm, *k* is the pooling ratio, and the selection of nodes is performed based on the average score *y* of the two head attention scores in the previous step in the following way, the top-*k* important nodes are selected based on the scores then the new node features and the new with a self-loop adjacency matrix are obtained. In the implementation of this project, *k* will be chosen 0.75 at last to facilitate the comparison with the original paper

9 idx=topk(Z,[kN])

10 $$X_{out}=X⊙Z_{idx};A_{out}=A_{idx}$$

#### 2.3.3 Mix Pooling Layer

In the first two layers of the convolutional module, due to the application of the self-attention graph pooling mechanism and top-*k* node selection based on graph and node features, the output obtained before the subsequent pooling step is primarily intended to reduce computational load and accelerate the training process, rather than directly affecting the graph features. The pooling method employed in this study is a combination of max pooling and sum pooling. The pooling ratio is set to 0.5, after which the two pooling results are concatenated to form the final output

11 $$X_{iout}=\frac{1}{N}\sum_{idx=0}^{N-1}X_{i}∥\max\left(X_{i}\right)$$

*X*_*i*out_ is the *i*th node feature, and we splice the max pooling result and the sumpooling result in the right side of this formula.

### 2.4 Evaluation

$$F_{\max}\left(\tau \right)=\max_{\tau \in \left[0,1\right]}\left\{\frac{2^{*}\mathrm{pr}{\left(\tau \right)}^{*}rc\left(\tau \right)}{\mathrm{pr}\left(\tau \right)+rc\left(\tau \right)}\right\}$$

To comprehensively assess the predictive performance of the proposed model, we employ several widely used evaluation metrics, including AUC, AUPR, and *F*_max_. These metrics provide complementary perspectives on the model’s ability to accurately predict protein functions. AUC (area under the receiver operating characteristic curve) evaluates the model’s ability to distinguish between positive and negative classes by computing the area under the ROC curve, which plots the true positive rate (TPR) against the false positive rate (FPR) across varying thresholds. A higher AUC value indicates better overall classification performance and stronger class separation. AUPR (area under the precision-recall curve): the precision-recall (PR) curve illustrates the trade-off between precision and recall at different decision thresholds. AUPR is particularly informative in settings with class imbalance, as it reflects the model’s ability to maintain high precision without sacrificing recall. *F*_max_ score represents the maximum F1-score achievable over all thresholds and balances both precision and recall. At a given threshold τ, it computes the mean precision (*pr*) and mean recall (*rc*) across the test set. *F*_max_ is defined as

12 $$F_{\max}\left(\tau \right)=\max_{\tau \epsilon \left[0,1\right]}\left\{\frac{2^{*}\mathrm{pr}{\left(\tau \right)}^{*}rc\left(\tau \right)}{\mathrm{pr}\left(\tau \right)+rc\left(\tau \right)}\right\}$$

13 $$\mathrm{pr}\left(\tau \right)=\frac{1}{m\left(\tau \right)}\sum_{i=1}^{m\left(\tau \right)}{\mathrm{pr}}_{i}\left(\tau \right)$$

14 $$rc\left(\tau \right)=\frac{1}{n}\sum_{i=1}^{n}{rc}_{i}\left(\tau \right)$$

where *m*(τ) denotes the number of proteins for which the predicted probability of at least one functional label is greater than or equal to threshold τ, and *n* is the total number of proteins in the test data set. Together, these metrics offer a robust evaluation of the model’s performance in multilabel protein function prediction, capturing both classification quality and sensitivity to label imbalance.

### 2.5 Training and Testing

To comprehensively evaluate the effectiveness of the proposed model, three experiments with distinct training configurations were conducted.

In the first experiment, we fully replicated the methodology, hyperparameters, and model architecture described in the Struct2GO paper.^23^ The training, validation, and test data sets for this experiment were obtained directly from the publicly available data set provided by the original authors.

In the second experiment, we introduced several modifications to the original pipeline. First, the ESM-2 model was used to generate protein sequence embeddings, replacing the previously used Seq2Vec embeddings. Additionally, one-hot sequence mappings were excluded from the node feature representation in the protein graph. Furthermore, the pooling strategy in the final pooling layer was altered: instead of the original approach, which applies max pooling twice followed by averaging, we used a combination of sum pooling and max pooling to better capture diverse structural signals. These changes were designed to allow a more direct and meaningful comparison of representational effectiveness.

The third experiment followed the same configuration as the second, with the only difference being the inclusion of one-hot encoded sequence features as part of the node feature input in the protein graph.

For the two experiments involving self-processed data, the data set was randomly partitioned into training, validation, and test sets in a 7:2:1 ratio. The core network architecture was organized into convolutional pooling blocks (ConvPoolBlocks), with each block consisting of a GraphConv layer, a SAGPool layer, and a mixed pooling layer.

After six stacked ConvPoolBlocks, the resulting outputs—consisting of the processed adjacency matrix and node features—were concatenated with the global sequence embeddings. This combined representation was then passed through a Transformer encoder composed of three layers and eight attention heads to further capture high-level interactions. The final output was processed by a three-layer fully connected classification module, which produced probability scores for multiple GO terms per protein.

To evaluate performance, we employed the metrics *F*_max_, AUC, and AUPR across all experiments. For the Struct2GO replication experiment, the original preprocessed data set and code provided by the authors were used for training and evaluation, ensuring comparability. Importantly, in the benchmark comparison section, we report the results from our own Struct2GO reproduction experiment rather than the original paper’s published results to maintain methodological consistency. For other comparative methods not directly evaluated against Struct2GO by its authors, we report the corresponding performance metrics as cited in relevant prior studies.

All experiments were conducted on data sets derived from the human proteome.

## 3 Results & Discussions

### 3.1 Performance Overview

Figure 2 presents ROC curves for the best-performing models on each GO category. The highest AUC values were achieved in the CCO (0.887), followed by MFO (0.836) and BPO (0.767), reflecting improved discrimination ability across all ontologies.

**Figure 2:**
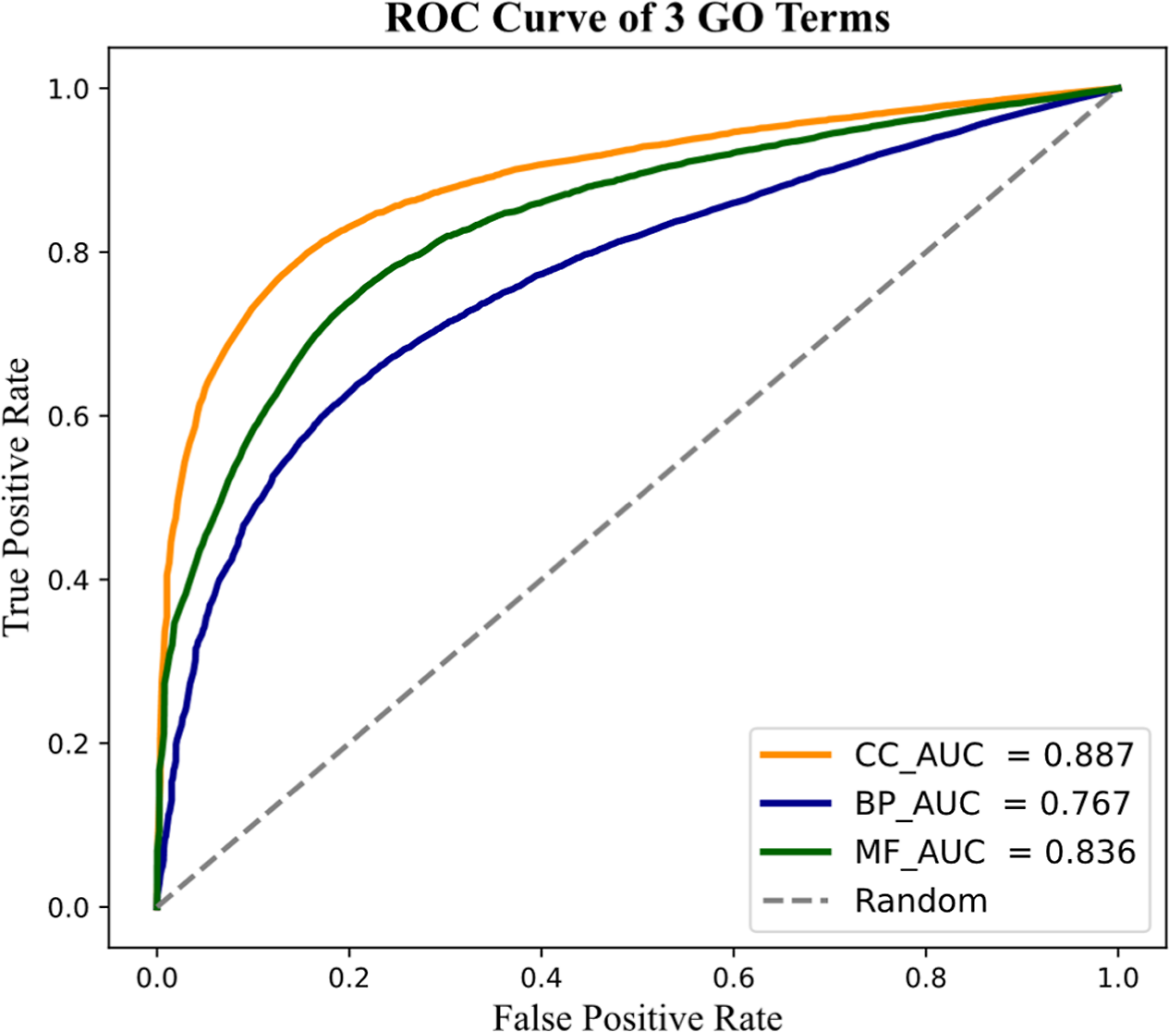
ROC curves of the best models.

Hyperparameter tuning revealed that a dropout rate of 0.2 and batch size of 32 yielded optimal performance for MFO and CCO, while a dropout of 0.3 and batch size of 64 were most effective for BPO. Notably, models incorporating one-hot residue features consistently outperformed those without them, reinforcing the benefit of combining high-resolution sequence embeddings with positional encoding in the graph input.

### 3.2 Comparative Analysis with Existing Methods

Table 1 summarizes the model performance across all GO categories and compares it against widely used baselines including BLAST, DeepGO,^11^ DEEPGOA,^28^ and the reproduced Struct2GO baseline.^23^ We have also performed McNemar tests to see the significant differences between our model and others. The results showed that we achieved better performance than others in most of measurement metrics.

**Table 1:** Model Performance Comparison on MFO, CCO, and BPO^a^

model	MFO			CCO			BPO		
	*F*_max_	AUC	AUPR	*F*_max_	AUC	AUPR	*F*_max_	AUC	AUPR
BLAST	0.339	0.577	0.489	0.441	0.563	0.269	0.411	0.623	0.461
DeepGO^11^	0.327	0.639	0.571	0.589	0.695	0.448	0.404	0.760	0.625
DEEPGOA^28^	0.385	0.698	0.622	0.629	0.757	0.5	0.477	0.820	0.71
Struct2GO^23^	0.302	0.808	0.256	0.531	0.886	0.601	0.303	0.749	0.261
without one-hot	0.384	0.836	0.418	0.501	0.867	0.483	0.250	0.725	0.194
with one-hot	0.399	0.836	0.421	0.557	0.887	0.600	0.334	0.767	0.305

a Struct2GO: reproduced result as baseline.

### 3.3 Effect of Proposed Enhancements

Our results demonstrate consistent performance gains across all GO categories when applying the two key enhancements.

- ESM-2 embeddings significantly outperform SeqVec, likely due to ESM-2’s deeper architecture and larger training corpus, which enable more effective capture of evolutionary and functional motifs. Prior work by Anteghini et al.^29^ showed that ESM-1b outperforms SeqVec in generating protein embeddings. As ESM-2 is a more advanced successor to ESM-1b, it was adopted in this study as a replacement for SeqVec with the expectation of improved performance—a hypothesis that was validated by our experimental results. These findings underscore the critical role of high-quality sequence feature extraction in advancing protein function prediction.
- Hybrid pooling (sum + max) enables richer aggregation of structural information compared to the original pooling design, contributing to performance stability.^30^
- One-hot residue encoding, when added to the graph node features, consistently boosts performance,^31^ particularly in MFO and BPO tasks where positional context appears more functionally informative.

In particular, for MFO, the *F*_max_ improved from 0.302 (Struct2GO) to 0.399, AUC increased from 0.808 to 0.836, and AUPR rose from 0.256 to 0.421. Similar trends were observed in CCO and BPO, highlighting the robustness of the enhancements. Even without the one-hot features, our ESM-2-based model outperforms Struct2GO in most metrics, underscoring the strength of upgraded sequence embeddings alone.

These results reinforce the hypothesis that multimodal learning, when designed carefully, can bridge gaps left by unimodal sequence or structure-based approaches. Our findings align with previous studies advocating for representation learning from both spatial and sequential protein features, but further push the boundary through improved architectural design.

### 3.4 Limitations and Future Work

While the proposed framework demonstrates notable performance gains, it inevitably incurs increased computational cost due to the integration of multiple modalities and model components. To mitigate this overhead, several strategies were adopted. First, we selected a moderate-sized variant of ESM-2, balancing representation quality with inference speed. Second, both ESM-2 sequence embeddings and Node2Vec-based structural embeddings were precomputed, reducing runtime during model training. Third, we constrained the depth of the graph convolutional and Transformer layers to prevent unnecessary model complexity. Collectively, these design choices reduced memory usage and training time, improving scalability and practicality for large-scale or cross-species protein function prediction.

Future work may focus on applying model compression techniques or developing lightweight architectures to further enhance computational efficiency without compromising predictive accuracy.

Despite the overall performance improvements across GO categories, the model exhibited relatively lower performance in BPO category, with reduced *F*_max_, AUC, and AUPR scores compared to the MFO and CCO categories. This limitation may stem from the inherent complexity and semantic heterogeneity of biological processes, which often involve multistep interactions and diverse functional contexts. Additionally, the label distribution in BPO is more imbalanced, with many rare terms underrepresented in the training data, making it more difficult for the model to learn informative patterns. Addressing this limitation may require class-aware training objectives or the integration of biological pathway knowledge to improve functional discrimination and representation within the BPO category.

## 4 Conclusion

We developed an enhanced graph-based framework for protein function prediction by integrating ESM-2 sequence embeddings, hybrid structural pooling, and one-hot residue encoding. Compared to the reproduced Struct2GO baseline, our model consistently improved performance across MFO, CCO, and BPO prediction, highlighting the benefit of combining advanced protein language models with refined graph-based strategies. These improvements are biologically meaningful, supporting more accurate functional annotation and potential drug target discovery. Although trained on human proteins, the framework’s design—featuring ESM-2 embeddings and residue-level representations—is inherently generalizable to other species, owing to the strong cross-species transferability of ESM-2. Future directions include expanding training to cross-species data sets, integrating PTM and protein–protein interaction data, exploring SE-equivariant GNNs, and optimizing computational efficiency through lighter ESM variants and graph sampling strategies. This work establishes a robust foundation for scalable, multimodal, structure-aware protein function prediction across diverse biological contexts.

## Data Availability

All data sets, models, and source code used in this study are publicly available at: https://github.com/khanhlee/esm-alphafold-go.
